# Citizen engagement in health policymaking: challenges and recommended solutions

**DOI:** 10.1093/eurpub/ckac131.539

**Published:** 2022-10-25

**Authors:** R Nakkash, R Abla, R Saleh, F El Jardali

**Affiliations:** 1 Health Promotion and Community Health, American University of Beirut, Beirut, Lebanon; 2 Health Management and Policy, American University of Beirut, Beirut, Lebanon; 3 Global and Community Health Department, George Mason University, Fairfax, USA

## Abstract

**Background:**

Involving citizens in health policymaking leads to the development of policies that are tailored to their needs and enables them to exercise their right as citizens. Citizen engagement in health policymaking in low and middle-income countries (LMICs) is rarely practiced and so understanding barriers and facilitators to engagement and the factors that impede effective participation is crucial. This study aims to understand views from policy stakeholders and citizens on citizen participation in health policy making and solicit recommendations on how to improve this process in the context of Lebanon.

**Methods:**

We conducted 29 individual in-depth interviews with stakeholders who work in fields relevant to health policy and/or have had experience in engaging citizens and four focus groups (average 8 to 10 participants each) with citizens from four municipalities in different governorates across Lebanon. Participants were purposively sampled through local Primary Healthcare Centers and municipality networks.

**Results:**

Barriers to engagement were seen as a manifestation of a dysfunctional and top-down political system, weak culture of participation, and lack of formal processes and platforms for engagement. Citizens’ attributed lack of participation to mistrust with the political system while on the other hand, stakeholders thought that citizens lacked the needed skills for active engagement. Recommendations for improvement focused on the importance of implementing system level changes, developing contextualized citizen engagement processes, and ensuring its adoption and implementation.

**Conclusions:**

Although participants identified many challenges to engagement, they acknowledged its value and were able to propose concrete solutions and recommendations for change. Those recommendations are useful for other LMICs of similar contexts whose mandates require participation.

**Key messages:**

• By identifying and understanding barriers to citizen participation in health policymaking, public health professionals can work towards improving engagement.

• Strategies and methods such as implementing system level changes and developing contextualized citizen engagement processes can be applied to improve citizen participation were needed.

